# Modulations of Cortical Power and Connectivity in Alpha and Beta Bands during the Preparation of Reaching Movements

**DOI:** 10.3390/s23073530

**Published:** 2023-03-28

**Authors:** Davide Borra, Silvia Fantozzi, Maria Cristina Bisi, Elisa Magosso

**Affiliations:** 1 Department of Electrical, Electronic and Information Engineering “Guglielmo Marconi” (DEI), University of Bologna, Cesena Campus, 47521 Cesena, Italy; davide.borra2@unibo.it (D.B.); mariacristina.bisi@unibo.it (M.C.B.); elisa.magosso@unibo.it (E.M.); 2 Interdepartmental Center for Industrial Research on Health Sciences & Technologies, University of Bologna, 40064 Bologna, Italy; 3 Alma Mater Research Institute for Human-Centered Artificial Intelligence, University of Bologna, 40121 Bologna, Italy

**Keywords:** electroencephalography, center-out reaching, event-related spectral perturbation (ERSP), event-related desynchronization (ERD), spectral Granger causality, in degree and out degree

## Abstract

Planning goal-directed movements towards different targets is at the basis of common daily activities (e.g., reaching), involving visual, visuomotor, and sensorimotor brain areas. Alpha (8–13 Hz) and beta (13–30 Hz) oscillations are modulated during movement preparation and are implicated in correct motor functioning. However, how brain regions activate and interact during reaching tasks and how brain rhythms are functionally involved in these interactions is still limitedly explored. Here, alpha and beta brain activity and connectivity during reaching preparation are investigated at EEG-source level, considering a network of task-related cortical areas. Sixty-channel EEG was recorded from 20 healthy participants during a delayed center-out reaching task and projected to the cortex to extract the activity of 8 cortical regions per hemisphere (2 occipital, 2 parietal, 3 peri-central, 1 frontal). Then, we analyzed event-related spectral perturbations and directed connectivity, computed via spectral Granger causality and summarized using graph theory centrality indices (in degree, out degree). Results suggest that alpha and beta oscillations are functionally involved in the preparation of reaching in different ways, with the former mediating the inhibition of the ipsilateral sensorimotor areas and disinhibition of visual areas, and the latter coordinating disinhibition of the contralateral sensorimotor and visuomotor areas.

## 1. Introduction

Planning goal-directed movements towards visual targets at different positions in space is at the basis of common daily activities (e.g., reaching, reach-to-grasping). The underlying neural processing mainly involves occipital, parietal, and frontal brain areas, spanning from visual to visuomotor to sensorimotor areas, reflecting movement preparation and initiation [1]. Specifically, movement preparation includes the perception of the visual cue, the extraction of high-level movement goals (e.g., a specific reach endpoint), and the computation of low-level movement commands [2,3].

Oscillations recorded with magneto- and electro-encephalography (M/EEG) describe the synchronous activity of thousands of anatomically aligned neurons. Neural oscillations are strongly modulated by motor tasks; during movement preparation and execution, the amplitude of M/EEG oscillations in alpha (8–13 Hz) and beta (13–30 Hz) bands is attenuated in the sensorimotor areas (post-central gyrus, pre-central gyrus, and supplementary motor areas) [4,5]. This phenomenon is known as event-related desynchronization (ERD) and is followed by a rebound, in general also exceeding the resting value, once the movement is executed (event-related synchronization, ERS) [4]. Such ERD can be interpreted as an electrophysiological neural correlate of activation (i.e., disinhibition) of cortical areas involved in processing motor-related sensory information or in the production of motor behavior [4], and was found to be modulated depending on the task complexity and performance [6,7,8]. ERD in sensorimotor regions starts up to 2 s before movement onset and, even when performing unimanual movements, does not remain confined in the hemisphere contralateral to the moved hand but also involves the ipsilateral hemisphere, both during movement preparation and execution [4,9,10]. The ERD ipsilateral to the moved hand was found to be modulated by task complexity [11], age [12], and pathology [13] and contributes to maintain the motor performance [14]. For example, in stroke patients, stronger alpha-ERD was observed in the ipsilateral central sites compared to contralateral ones while moving their paretic hand [13], supporting the idea that ipsilateral sensorimotor activity may compensate deficits related to pathology to preserve motor performance.

Besides contralateral and ipsilateral sensorimotor areas, other areas are also involved in motor control depending on the motor task, such as parietal and occipital areas, both in the contralateral and ipsilateral hemisphere [14,15]. Therefore, it is well established that successful motor functioning depends on the interactions and communications among multiple brain regions [16]. Understanding how these areas interact is crucial from a neurophysiological perspective, to gain insights into the mechanisms underlying motor functions, both in healthy subjects and in patients. This knowledge can also be instrumental for diagnostic applications and for the development of assistive and rehabilitation devices. Indeed, brain connectivity analysis during motor tasks is a topic of intense investigation in neuroscience, using both functional Magnetic Resonance Imaging (fMRI) techniques and M/EEG techniques. The former are characterized by high spatial resolution allowing a more precise anatomical allocation of connectivity couplings, but have poor temporal resolution. The latter have a coarser spatial resolution, but their high temporal resolution allows connectivity to be characterized in specific frequency bands, thus examining how brain interactions are associated with different, functionally relevant brain rhythms. In EEG-based studies, patterns of connectivity related to motor tasks are often analyzed in alpha and beta bands (e.g., see [17,18,19,20]), although connectivity in other spectral ranges (gamma, i.e., >30 Hz, delta, i.e., 1–4 Hz, theta, i.e., 4–8 Hz) is sometimes investigated too (e.g., see [21,22,23]). In the following section, some results of connectivity studies (both fMRI- and M/EEG-based) are delineated.

The activity of ipsilateral sensorimotor regions appears to be modulated via inter-hemispheric interactions from the contralateral hemisphere [14,16,24,25]. While the exact role of ipsilateral sensorimotor areas and of the connectivity coupling with the contralateral ones is still debated, results corroborate the view that these mechanisms participate somehow to control and perform unimanual movements [26]. Indeed, evidence was found about inter-hemispheric interactions promoting inhibition in the ipsilateral sensorimotor areas, to facilitate the contralateral processing of an upcoming movement. Interestingly, in stroke, inhibitory influences appear decreased from the sensorimotor regions of the lesioned hemisphere towards the contralesional ones; this suggests that a motor network reorganization takes place so that the contralesional regions (ipsilateral to the affected hand) may help the movement of the affected hand [20]. Oscillations in the alpha band have been suggested to mediate a general inhibitory mechanism helping in the suppression of task-irrelevant or task-interfering information [27,28]. An alpha-mediated inhibitory mechanism was observed while planning actions (externally triggered), as the increase in inter-hemispheric sensorimotor connectivity in the alpha band was found to inhibit more the ipsilateral sensorimotor areas [10]. Moreover, inter-hemispheric coupling between sensorimotor areas was found strengthened in the alpha and beta bands when task complexity increases and when learning new motor programs [26]. Thus, increase in bilateral interactions has been also associated to increased exigencies on the motor system [26].

Furthermore, motor tasks have been found to encompass connectivity changes in largely distributed networks involving areas beyond the central sensorimotor ones. In particular, there is vast evidence that fronto-central sensorimotor cortices and posterior parietal cortices are cooperatively involved in goal-directed actions (e.g., reaching, grasping, interacting with objects and tools) to dynamically integrate sensory inflows and motor outflows, for movement planning and selection, and online monitoring [29,30,31]. In this regard, an increased connectivity between parietal and motor cortices was observed in the beta band during lever pressing [32] and during preparation and execution of praxis hand movements [33]. Lastly, beta-band connectivity between parietal and motor cortices was also found to be modulated by the amount of visual information in a visuo-motor reaching task [34].

Despite the intense research and the large amount of collected findings, some aspects remain under-investigated. In particular, although reaching is a key component of motor actions that allow humans to interact with the environment, only a limited number of studies have examined EEG-based connectivity in reaching tasks [23,34,35]. Chung et al. [34] investigated EEG oscillatory activity and directed connectivity (via dynamic causal modeling) during the execution of visually-guided ballistic arm movement, and compared the effect of high vs. low visual gain. The study focused on two cortical areas (left sensorimotor and medial parietal), considered the movement and post-movement phase, and characterized connectivity differences between movements in the two visual conditions. Caliandro et al. [23] analyzed scalp ERD/ERS during the execution of reach and grasp movements, and quantified changes in the source-level connectivity network over the whole cortex during movement compared to rest; they used a non-directed connectivity measure (lagged coherence), and applied a graph analysis to evaluate the ‘small world-ness’ property of the network. From MEG data, Yeom et al. [35] applied a time-window shifting approach to explore changes in brain connectivity with motor states, from movement preparation to movement execution; non-directed connections (using mutual information) were estimated between motor-related cortical regions, and graph-based degree centralities were computed to identify network hubs. While these studies of course provide relevant results, they suffer from the limitations that either non-directed metrics of connectivity were used to investigate couplings among several widely distributed regions [23,35] or directional metrics were used to investigate the coupling between two brain regions only [34]. Thus, a description is desired about the frequency-specific changes in directional-dependent interactions evoked by a reaching task within a large network of task-related areas, including occipital (visual), parietal, and fronto-central cortices.

In this study, we aim at contributing to this description by investigating alpha- and beta-band oscillatory mechanisms in key brain regions during *reaching movement preparation* at two levels of analysis: (i) modulations of regional power (ERD/ERS), as measured by event-related spectral perturbations; (ii) changes in interactions between brain regions, as measured by a directed connectivity measure (spectral Granger causality). To this aim, we recorded EEG from 20 healthy participants while performing a delayed center-out reaching task towards five different positions equally spaced and located in a semi-circle. The EEG activity was projected to the cortex, and the activity of 16 cortical regions of interest (ROIs), 8 per hemisphere, was considered, by selecting the ROIs most involved in the planning and control of reaching movements. First, a time-frequency analysis was conducted to reveal the event-related spectral perturbations associated with reaching movement preparation, focusing on the alpha and beta bands. Then, directed connectivity between the ROIs in the alpha and beta bands was analyzed via spectral Granger causality. Differences in the connectivity network between reaching preparation and rest (baseline) were emphasized using two indices derived from the graph theory, i.e., the in degree and out degree centrality indices quantifying the overall connectivity inflow and outflow for each ROI.

## 2. Materials and Methods

### 2.1. Participants

Twenty healthy volunteers (11 M and 9 F, aged 21.9 ± 2.3 years, mean (m) ± standard deviation (std)) participated in the study. They were all right-handed and had normal or corrected-to-normal vision. The study was approved by the Bioethics Committee of the University of Bologna (protocol code: 61243, date of approval: 15 March 2021) and written informed consent was obtained from all participants before the beginning of the experiment. All data were analyzed and reported anonymously.

### 2.2. Experimental Protocol and EEG Data Acquisition

**Figure 1:**
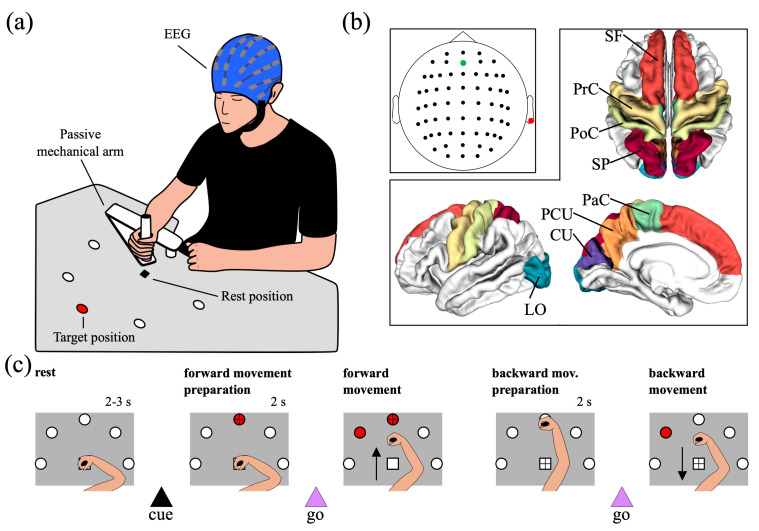
(**a**) Schematics of the recording setup. (**b**) Location of electrodes and regions of interest (ROIs). The electrodes were placed according to the 10/10 system and the ROIs considered in this study were taken from the Desikan–Killiany atlas. The reference channel (right earlobe) is marked in red, while the ground channel (AFz) in green. The selected ROIs were the cuneus (CU) and lateral occipital (LO) cortices as occipital regions, the precuneus (PCU) and superior parietal (SP) cortices as parietal regions, the post-central gyrus (PoC), the precentral gyrus (PrC), and the paracentral lobule (PaC) as peri-central regions, and the superior frontal gyrus (SF) as frontal region. (**c**) Trial sequence. Each trial started with a rest interval (2–3 s, random) that ended once a cue signal (target LED turning on) was provided to the participant indicating the target position. Then, the participant started preparing the center-out reaching movement (forward movement) and started the movement only after 2 s, once the first go-signal was provided (neighbor LED turning on). Once they reached the target, the participant held the position for 2 s while all LEDs were turned off. Finally, the second go-signal was provided (same as for the forward movement), triggering the backward movement toward the rest position. The fixation cross is displayed for each interval; note that, in the first scheme of panel c (rest interval 2–3 s), the fixation cross (at the rest position) is not visible since covered by the participants’ hand.

The experimental paradigm consisted of a delayed center-out reaching task towards five different positions equally spaced and located in a semi-circle (see Figure 1a). The reaching targets were five red LEDs placed on a wooden plane (i.e., reaching was performed in 2-D). LEDs were controlled using a DAQ NI USB-6008 board (National Instruments Corp., Austin, TX, U.S.) controlled via MATLAB^®^ (The Mathworks Inc., Natick, MA, USA). The participants sat upright in front of the semi-circle. To support the participants’ arm and to reduce the participants’ fatigue during the task, the task was performed with their right arm on top of a custom-made passive mechanical arm with 2 joints (see Figure 1a), sliding over the plane by means of a rolling ball bearing.

The experimental session consisted of 6 blocks, with a short break between blocks. In each block, 50 trials were acquired, reaching one of the 5 targets in each trial (10 repetitions for each target). The sequence of targets to reach was randomly generated in each block. Each trial started with the participants’ hand resting at the center of the semi-circle (*rest position*) while the participant fixated on the center of the semi-circle. After a random interval between 2 and 3 s (rest interval) sampled from a uniform distribution, one of the five LEDs turned on, representing the target to reach (cue-signal, *target position*). Then, the participant fixated on the target to reach, waiting for 2 s for the go-signal. The go-signal was provided by turning one of the LEDs adjacent to the LED to reach. Once the go-signal was provided, the participant started the reaching movement towards the target (forward movement, corresponding to a 2-D center-out reaching movement), and once they reached the target, both LEDs providing the cue-signal and go-signal turned off. Then, the participant switched the fixation from the target LED back to the center of the semi-circle and remained at the target for 2 s. Finally, a new go-signal was provided by turning on the same LED used as go-signal in the forward movement, and the participant started moving back to the rest position (backward movement).

At the beginning of the session, each participant wore an EEG cap with 61 electrodes (1 passive (ground) + 60 active g.SCARABEO electrodes, g.tec Medical Engineering GmbH, Schiedlberg, UA, Austria) placed according to the 10/10 system. The reference electrode was placed on the right earlobe and the ground electrode in AFz (see Figure 1b for electrode locations). Signals were amplified with g.HIAMP80 Research amplifier (g.tec Medical Engineering GmbH, Schiedlberg, UA, Austria), sampled at 512 Hz, and electrode impedances were kept below 50 kΩ. A notch digital filter (stopband of 48–52 Hz), performed by the digital signal processor of g.HIAMP80, was applied during recording.

### 2.3. EEG Data Analysis

In this study, the analysis was focused on source-level changes in EEG activity and connectivity that occurred during the interval of preparation of the forward movement (from the center to a periphery point), i.e., between the cue-signal (black triangle in Figure 1c) and the first go-signal (first violet triangle in Figure 1c).

#### 2.3.1. EEG Pre-Processing

The pre-processing consists of the following steps:

- Linear detrending of signals belonging to each recording block.
- Band-pass filtering between 1 and 60 Hz and notch filtering at 50 Hz of signals belonging to each recording block. Notch filtering was applied also offline since visualization of the power spectral density of the recorded EEG signals evidenced insufficient attenuation of the power line noise by the notch filter applied during recording.
- Identification of bad channels within signals of each recording block via random sample consensus method (RANSAC) [36].
- Concatenation of electrode signals across recording blocks.
- Removal of channels that were labelled as bad (step iii) at least in one recording block (3 ± 2 channels per subject removed, m ± std, ranging from 0 to 8).
- Removal of artifacts (ocular, muscular, heart, and channel noise) via independent component analysis (ICA) via visual inspection of the components. ICA was computed using the extended Infomax algorithm [37,38] which estimates mixed sub-Gaussian and super-Gaussian sources. Across subjects, 31 independent components were removed, on average (ranging from 25 to 39). This relatively large number of removed ICs derives from the long-lasting recording (3750 s overall, obtained by concatenating 6 blocks) and on the type of performed task. Indeed, tasks involving motor activities are more prone to create isolated non-stereotypic artifacts (such as electrode pops, or complex movement artifacts) that are extracted in separated ICs and that add to the classical ICs separating stereotypic artifactual activity such as blinking, eye-movements, and heartbeat. We visually explored each IC (its time pattern, power spectral density, and topological map) carefully before removing it, in an effort to minimize the removal of potentially useful activity.
- Spherical spline interpolation of the bad channels removed in step v.
- Epoching into 4 s-length trials, starting 1 s before and ending 3 s after the presentation of the cue-signal, i.e., after the target LED to reach turned on. Thus, trials were defined from −1 s to 3 s, where 0 s corresponds to the onset of the cue-signal (corresponding to the black triangle in Figure 1c).
- Baseline correction of each trial, by removing the mean value computed over the rest interval from −1 s to 0 s, channel by channel.
- Common average re-referencing.

All pre-processing steps were performed in Python using custom scripts and the functionalities of MNE Python library (version 1.2.2) [39] for implementing step vi.

#### 2.3.2. Cortical Activity Reconstruction and Computation of Activity within Regions of Interest (ROIs)

Sensor-space signals (scalp signals) were transformed into source-space signals (cortical signals) using MNE Python library (version 1.2.2) [39]. A template head anatomy was adopted using the FSaverage template, with the source space restricted to the cortex and discretized into 20,484 vertices. The forward problem [40] was solved via the boundary element method, applying MNE default parameters. The inverse problem [41] was solved using eLORETA (exact Low-Resolution Electromagnetic Tomography) [42] with MNE default parameters, with identity noise covariance matrix, and with the dipole source orientation constrained to be perpendicular to the cortex, resulting in one source signal per cortical vertex (i.e., 20,484 source signals).

The whole cortical surface was parcellated into 68 regions according to the Desikan–Killiany atlas [43], and we selected 8 regions of interest (ROIs) per hemisphere (16 in total) for our analysis. The selection of the ROIs was based on a priori information, considering the regions reported in the literature as most involved in motor planning and control during reaching movements [15,44,45]. The selected ROIs (see Figure 1b) included:

- The cuneus (CU) and the lateral occipital cortex (LO), located in the occipital lobe; they mainly have visual functions.
- The precuneus (PCU) and the superior parietal lobule (SP), located in the posterior parietal cortex; they have associative (mainly visuomotor) functions, and their activations has been specifically associated to planning and execution of reaching movements [29].
- The post-central gyrus (PoC), the precentral gyrus (PrC), and the paracentral lobule (PaC), located in the peri-central part of the cortex. They include the somatosensory cortex (PoC), the primary motor, premotor, and supplementary motor areas (PrC and PaC), and overall are denoted as sensorimotor ROIs.
- The superior frontal gyrus (SF), located in the frontal region and implicated in high-level motor control functions [46].

For each trial, a single waveform representative of the neural activity of each ROI was derived, by averaging all signals of the vertices belonging to that ROI. To avoid cancelling out the activity in case of many vertices in the ROI with dipole orientations in opposite directions, the signs of source signals that were not oriented as the “dominant direction” were flipped before averaging, as performed in Ghumare et al. [47]. The dominant direction was the first principal direction of all dipole orientations within the ROI. This sign flip procedure is adopted by Brainstorm toolbox [48] when using constrained dipole orientations.

#### 2.3.3. Cortical Event-Related Spectral Perturbation

For each subject, each trial, and each ROI, the cortical event-related spectral perturbation (ERSP) were obtained based on the continuous wavelet transform of the cortical signal representative of that ROI using complex Morlet wavelet as basis function. Specifically, ‘cmor1.5-1.0′ was used as mother wavelet, with the first parameter denoting the bandwidth and the second parameter the normalized center frequency (normalized by the sampling period) [49]. Therefore, the mother wavelet had center frequency of 512 Hz with 4 oscillations (scales from 64 to 42 for alpha band, and from 42 to 16 for beta band). The wavelet transform coefficients were squared to obtain time-frequency power representations. Then, for each subject and each ROI, these representations were averaged across trials, separately for each of the 5 target positions, and normalized using the rest interval between −1 and 0 s as baseline (see Grandchamp et al. [50]). Specifically, for each frequency, the average power value between −1 and 0 s was computed, obtaining the average baseline power frequency by frequency. Then, the *ERSP* was computed as the difference between the power at each time-frequency point and the average baseline power at the same frequency, divided by this same average baseline power (thus, *ERSP* expresses the difference with respect to the baseline in percentage of the baseline).

Subsequently, for each subject and each ROI, the *ERSP* was averaged over the alpha band (8–13 Hz) and beta band (13–30 Hz), obtaining *alpha-ERSP* and *beta-ERSP*. We performed preliminary analyses by testing differences in *alpha-ERSP* and in *beta-ERSP* across different targets via permutation cluster tests [51] between each possible pair of targets, corrected for multiple comparisons via Benjamini–Hochberg [52] false discovery rate for each band. As no significant difference was found (p>0.05), *alpha-ERSP* and *beta-ERSP* were also averaged across targets. Lastly, the time interval between the cue-signal and the go-signal was divided into two non-overlapped 1 s-length windows, i.e., from 0 to 1 s (early post-cue window) and from 1 to 2 s (late post-cue window, hereafter referred as *post-cue_late_*). Then, we averaged the *alpha-ERSP* and *beta-ERSP* over the late post-cue window, obtaining the *post-cue_late_ alpha-ERSP* and *post-cue_late_ beta-ERSP*, which were assumed as mainly representative of the alpha and beta perturbations related to reaching movement preparation. The choice of considering this window is justified since the ERSPs in the early post-cue window were strongly affected by the visual evoked potential elicited by the lighting of the target LED.

This analysis was performed using custom Python scripts and the Python library PyWavelets [53] (version 1.4.1).

#### 2.3.4. Cortical Functional Connectivity and Degree Centralities (in Degree, out Degree)

For each subject, directional influences between ROIs in alpha and beta bands were estimated by computing pairwise Granger causality (GC) [54] in the frequency domain. Denoting with $x_{i}[n]$ and $x_{j}[n]$ two time series, here corresponding to the cortical signals representative of the i-th and j-th ROI (see Section 2.3.2), the system $\left[x_{i}[n];x_{j}[n]\right]$ can be represented using a bivariate autoregressive model with order p (p=30 in this study, as we already adopted in previous studies, e.g., in Magosso et al. [55]). By Fourier-transforming this time-domain representation, a spectral representation is obtained. Then, the power spectrum of each time series (e.g., $x_{i}[n]$) can be computed according to Geweke [56] and decomposed into an intrinsic term and a causal term, the latter being the term predicted by the other time series (e.g., $x_{j}[n]$). The spectral GC from the j-th to the i-th ROI at each frequency f, ${GC}_{j\to i}\left(f\right)$, is defined as the log of the ratio between the total power of $x_{i}[n]$ at f and the difference between the total power of $x_{i}[n]$ at f and the causal power exerted by $x_{j}[n]$ onto $x_{i}[n]$ at f. Thus, the quantity ${GC}_{j\to i}\left(f\right)$ increases as the causal power increases. At each frequency f, the spectral GC is represented by a non-symmetric matrix with shape $N_{ROI}{\times N}_{ROI}$ ($N_{ROI}=16$ in this study), with the off-diagonal ji-th value quantifying the directional influence from the j-th ROI to the i-th ROI at that frequency (${GC}_{j\to i}\left(f\right)$).

Spectral GC was computed separately within two different 1 s-length windows, i.e., the baseline (rest) window from −1 to 0 s and the late post-cue window (reaching movement preparation, *post-cue_late_*) from 1 to 2 s. The window from 0 to 1 s was neglected since it was strongly influenced by the transient due to the visual event related potential, elicited by the cue-signal. Still, to compensate for residual non-stationarities that might occur also in the considered windows in case of non-complete exhaustion of the evoked potential in the first post-cue second, the evoked potential was removed from each trial [57]. Rest windows and movement preparation windows were concatenated across trials, and spectral Granger causality was computed over the concatenated trials, thus estimating directional influences between ROIs across all trials in the two conditions. *Alpha*-GC and *beta-GC* were computed by averaging together the values of the GC spectrum belonging to alpha and beta bands, separately in the baseline and late post-cue conditions, resulting in 4 total connectivity matrices ($A\in R^{N_{ROI}{\times N}_{ROI}}$) per subject (*baseline alpha-GC*, *baseline beta-GC*, *post-cue_late_ alpha-GC*, *post-cue_late_ beta GC*). Furthermore, each connectivity matrix was normalized such that the sum of all off-diagonal connectivity values was 1 ($\tilde{A}=A/\sum_{i,j;i\neq j}A_{ij}$), thus emphasizing how much each connectivity value contributed to the overall connectivity across the selected ROIs as performed in [20].

Finally, indices derived from the graph theory were used to better understand changes in the topology of the brain connectivity network between baseline (rest) and late post-cue (movement preparation) conditions. Indeed, each matrix containing the connections values between the ROIs can be represented as a weighted directed graph, where each node corresponds to an ROI and the weight of each directed edge corresponds to the connection value. We computed two centrality indices, taking into account the direction of connections: the *in degree*—i.e., the sum of connectivity values entering into each ROI (quantifying the overall connectivity inflow)—and the *out degree*—i.e., the sum of connectivity values departing from each ROI (quantifying the overall connectivity outflow). The two indices were computed for each connectivity matrix, i.e., for each band (alpha and beta) and each condition (*baseline*, *post-cue_late_*).

This analysis was performed using custom Python scripts, replicating the functions of the Brainstorm toolbox [48] (version 3.221212) that compute spectral Granger causality.

### 2.4. Statistical Analyses

The following tests were conducted:

- For all 16 ROIs, *post-cue_late_ alpha ERSP* and *post-cue_late_ beta ERSP* were compared to 0 (corresponding to the average baseline value after normalization), using Wilcoxon signed-rank tests. This comparison was performed to identify ROIs with a different ERSP during movement preparation compared to rest, separately for alpha and beta (16 test for each band). To correct for multiple tests, false discovery rate correction at α=0.05 was applied, using the Benjamini–Hochberg procedure [52].
- For all pairs of homologous ROIs, *post-cue_late_ alpha ERSP* and *post-cue_late_ beta ERSP* were compared between left and right ROIs using Wilcoxon signed-rank tests. This comparison was applied to identify ROIs with a lateralization in the spectral perturbations, separately for alpha and beta (8 tests, for each band). To correct for multiple tests, false discovery rate correction at α=0.05 was applied, using the Benjamini–Hochberg procedure [52].
- *Alpha-GC* and *beta-GC* were compared between *post-cue_late_* and *baseline* using permutation tests (5000 permutations) [51]. This was performed to identify connections between ROIs that resulted in significantly differences during movement preparation compared to rest, separately for alpha and beta (16*15 = 240 tests for each band).
- For all ROIs, *in degree* and *out degree* in each band were compared between *post-cue_late_* and *baseline* using Wilcoxon signed-rank tests. This was performed to identify ROIs with a different inflow or outflow during movement preparation compared to rest, separately for alpha and beta (16 tests for each band and centrality index). To correct for multiple tests, false discovery rate correction at α=0.05 was applied, using the Benjamini–Hochberg procedure [52].

## 3. Results

### 3.1. Cortical Event-Related Spectral Perturbation

**Figure 2:**
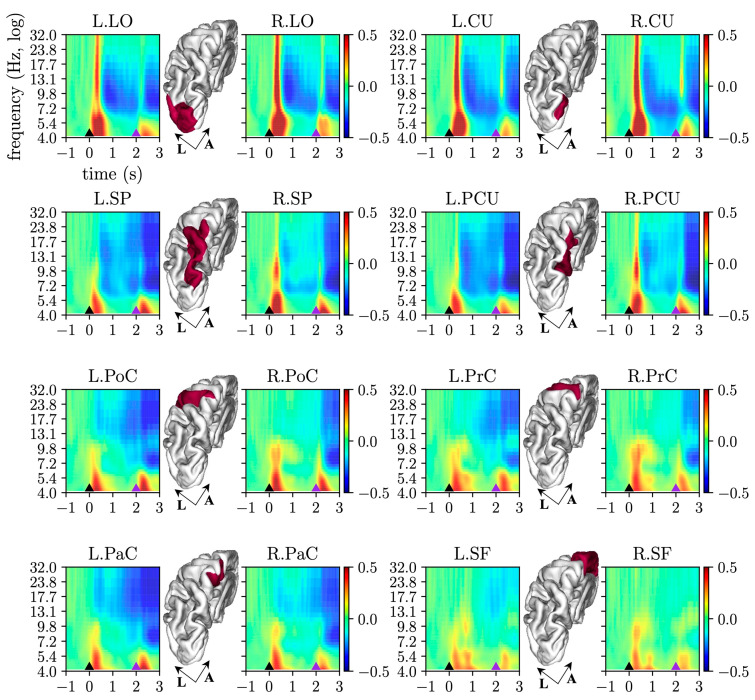
Event-related spectral perturbations (ERSPs). The grand-average ERSP is reported for each selected ROI of the left (label prefix “L.”) and right (label prefix “R.”) hemisphere. The small black and purple triangles at the bottom of each plot mark the time associated with the cue onset and go onset of the center-out reaching movement, respectively. To increase readability, x- and y-labels are reported only for the first plot. The position of each ROI is also visualized, limited to the left hemisphere, highlighted in red in the 3-D view of the cortex (A: anterior, L: lateral).

The grand average *ERSP* for each ROI is reported in Figure 2. A strong ERS is evident, spanning from the theta band (4–8 Hz) to the low beta band, associated to the visual evoked potential elicited by the cue-signal and go-signal (LED turning on). The ERS extinguished approximately 500 ms after each stimulus onset denoted by the black (cue) and purple (go) triangle in Figure 2. As expected, the ERS resulted more pronounced in visual (LO, CU) and visuomotor ROIs (SP, PCU) compared to the other ROIs, due to the visual nature of the stimuli. Furthermore, a clear ERD can be observed during the movement preparation period, in particular from 0.5–0.6 s to 2 s and during movement too, i.e., after the go-signal (from 2 to 3 s). The ERD involves both alpha and beta bands in the visual (LO, CU) and visuomotor ROIs (SP, PCU), and especially the beta band in the sensorimotor ROIs (PoC, PrC, PaC). Finally, the most frontal ROI included in the analysis (SF) showed less ERD compared to other ROIs.

**Figure 3:**
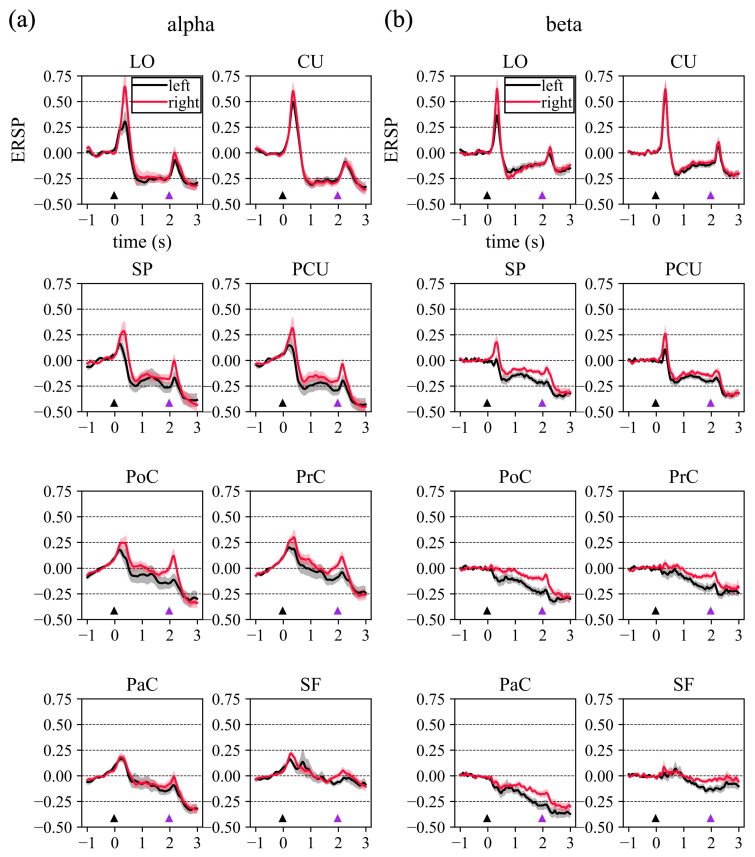
Alpha (**a**) and beta (**b**) event-related spectral perturbations (ERSPs). Here, the ERSPs reported in Figure 2 were averaged within alpha and beta bands and visualized as a function of time. The grand-average alpha-ERSP and beta-ERSP is reported for each selected ROI of the left (black thick lines) and right (red thick lines) hemisphere. Shaded areas denote the standard error of the mean across subjects (in grey for the left ROI, in red for the right ROI). The small black and purple triangles shown at the bottom of each plot mark the time associated with the cue onset and go onset of the center-out reaching movement, respectively. Note that in this figure, to increase the readability, x- and y-labels are reported only for the first plot.

By averaging the ERSPs represented in Figure 2 over the alpha and beta bands, the *alpha-ERSPs* and *beta-ERSPs* were computed and are reported in Figure 3, to better visualize the ERSP temporal dynamic in these frequency ranges. Concerning *alpha-ERSPs* (Figure 3a), the following observations can be made with a focus on the movement preparation period (i.e., 0–2 s). In agreement with Figure 2, a strong ERS was elicited by the cue-stimulus, especially in visual (LO and CU) and visuomotor ROIs (PCU and SP). In the other ROIs, ERS was smaller. Cue-related ERS was followed by ERD (except than in SF), especially in the late post-cue interval (1–2 s). In this interval, ERD was approximately constant in visual and visuomotor ROIs, while it kept gradually increasing (from approximately 0 to −15%) in the sensorimotor ROIs (PoC, PrC, PaC).

Concerning *beta-ERSPs* (Figure 3b) in the same period (0–2 s), similar observations held in visual and visuomotor ROIs, with evident ERS produced by the cue stimulus followed by ERD, in particular in the late post-cue interval. In the other ROIs, only ERD occurred. Furthermore, the pattern of beta-ERD exhibited some differences compared to alpha-ERD. In the sensorimotor ROIs, the gradual increase in post-cue ERD was more pronounced in the beta band (up to −25%) than in the alpha band. Furthermore, in several ROIs, beta-ERD showed appreciable differences between the two hemispheres, with the contralateral hemisphere reaching lower ERD values (up to −25%) compared to the ipsilateral hemisphere (up to −12%). Finally, while in visual ROIs alpha-ERD was almost constant during the late post-cue interval, in the same interval, beta-ERD in the visual ROIs tended to decrease (i.e., assumed less negative values), showing a partial return towards baseline value (i.e., 0%).

Of course, as the reported representations refer only to epochs including rest (from −1 to 0 s), reaching preparation (from 0 to 2 s), and at least part of center-out reaching execution (from 2 to 3 s), the rebound of the ERSP recovering the resting condition value before the start of the new trial (i.e., 0) is not evident from these figures. Thus, Supplementary Figure S1 displays the alpha- and beta-ERSP over a longer epoch for Cz, an electrode site representative of the motor-related response, showing that the ERSP rebounded once the subject returned to the rest position (backward movement completed), and confirming that resting condition is recovered before the beginning of a new trial.

**Figure 4:**
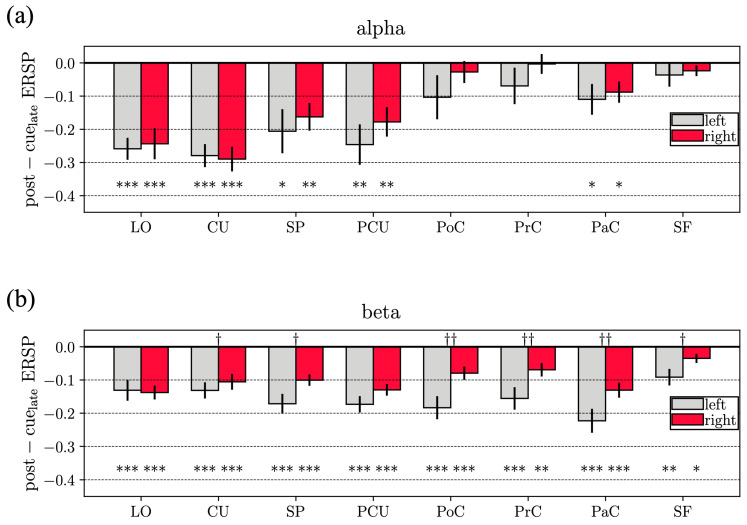
Alpha (**a**) and beta (**b**) event-related spectral perturbations (ERSPs) during reaching movement preparation. Here, the alpha-ERSP and beta-ERSP reported in Figure 3 were averaged within the second half of the movement preparation interval of the center-out reaching movement (i.e., from 1 to 2 s with respect to cue onset). These values are also referred to in the manuscript as *post-cue_late_ alpha-ERSP* and *post-cue_late_ beta-ERSP*. In each panel, for each ROI (grey: left ROI, red: right ROI) the bar height denotes the mean value across the subject and the error bar the standard error of the mean. Results of the performed statistical analyses are reported too. Specifically, symbols * (reported at the bottom of each panel) denote ERSPs significantly different compared to the baseline (* p<0.05, ** p<0.01, *** p<0.001). Symbols † (reported at the top of each panel) denote ROIs with significantly different ERSP between the left and right hemisphere († p<0.05, †† p<0.01).

Figure 4 reports the alpha- and beta-ERSP averaged over the late post-cue interval (*post-cuelate alpha-ERSP* and *post-cuelate beta-ERSP*), and the results of the statistical analyses. Significant ERD (p<0.05) during movement preparation was obtained for all ROIs compared to rest in the beta band, and for visual and visuomotor ROIs in the alpha band. Furthermore, ERD results were significantly (p<0.05) stronger in the contralateral hemisphere in the beta band (but not in the alpha band) for all ROIs except LO and PCU, with higher significance for sensorimotor ROIs (p<0.005 for PoC and PaC, p<0.01 for PrC).

### 3.2. Cortical Functional Connectivity and in Degree and out Degree Indices

**Figure 5:**
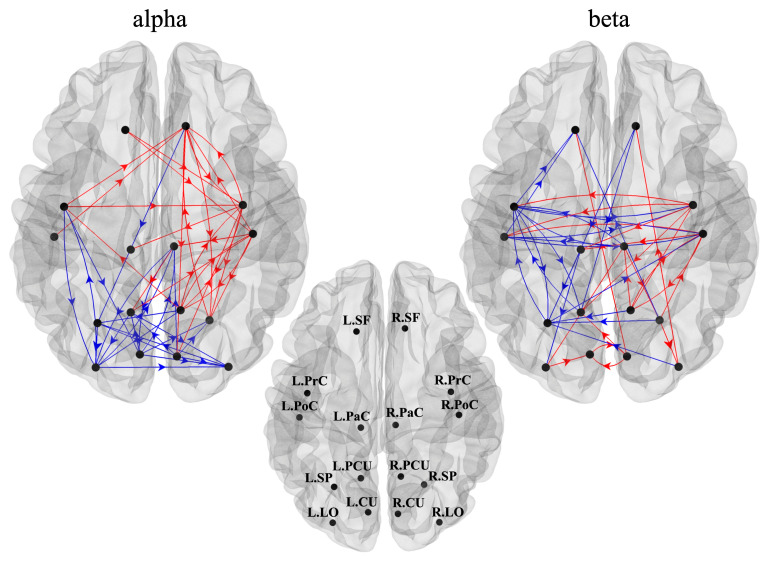
Directed connections between ROIs—as measured by the spectral Granger causality—that resulted significantly higher (in red) or lower (in blue) during reaching movement preparation compared to rest, in alpha (**left**) and beta (**right**) bands. To improve readability, ROI labels are displayed on the cortex in the middle panel, separately from the other panels.

The connections that were significantly higher (in red) or lower (in blue) in the late post-cue interval compared to baseline are displayed in Figure 5, separately for the alpha band (left panel) and beta band (right panel). Decreased alpha-band connectivity was mainly localized posteriorly, involving bilateral visual (occipital) and visuomotor (parietal) ROIs, but also with a left-lateralized involvement of sensorimotor regions (L.PrC). Increased alpha-band connections were mainly directed from left to right, especially toward right sensorimotor regions. As to the beta band, left ROIs (in particular visuomotor and sensorimotor) exhibited decreased connections, both entering and exiting, while right ROIs overall showed increased entering and exiting beta-band connections.

**Figure 6:**
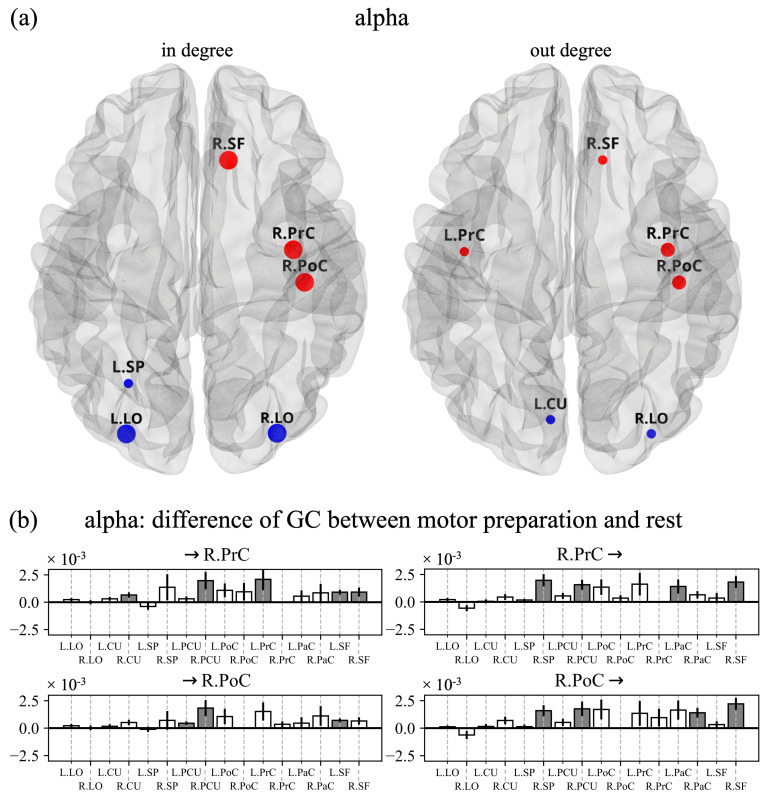
(**a**) ROIs with a significantly different in degree (left panel) and out degree (right panel) during reaching movement preparation compared to rest in the alpha band. Circle size reflects the strength of the significance (small: p<0.05, medium p<0.01, large: p<0.001); red/blue circles denote an increased/decreased measure (in degree or out degree) during movement preparation compared to rest. (**b**) Each bar plot shows, for a selected ROI among the ones in panel a, the difference in the connections (movement preparation—rest) entering in the selected ROI from all other ROIs or exiting from the selected ROIs towards all other ROIs. The bar height denotes the mean value across the subjects and the black line the standard error of the mean. Significant differences are marked via grey bars.

**Figure 7:**
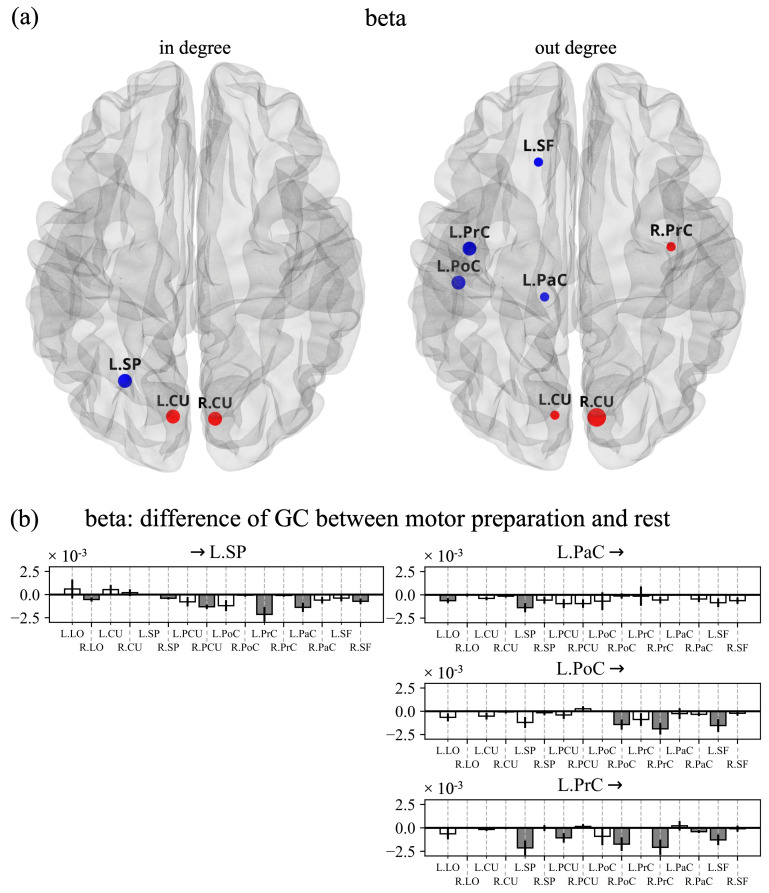
(**a**) ROIs with a significantly different in degree (left panel) and out degree (right panel) during reaching movement preparation compared to rest in the beta band. (**b**) Each bar plot shows, for a selected ROI among the ones in panel a, the difference in the connections (movement preparation—rest) entering in the selected ROI from all other ROIs or exiting from the selected ROIs towards all other ROIs. See the caption of Figure 6 for further details.

Figure 6a and Figure 7a report the ROIs that exhibited significantly different in degrees (left) and out degrees (right) for the alpha and beta bands, respectively, when comparing late post-cue interval to baseline. Each bar plot in Figure 6b and Figure 7b shows, for a selected ROI, the difference (late post-cue minus baseline) in the connections entering into the selected ROI from each other ROI, or exiting from the selected ROI towards each other ROI. The shown differences highlight the ROIs contributing more to the in degree or out degree of the selected ROI (significant differences are indicated by grey bars).

As to the alpha band (Figure 6), the late post-cue interval was characterized by a significantly lower alpha-inflow in bilateral visual ROIs (L.LO and R.LO), a significantly higher alpha-inflow in ipsilateral frontal ROI (R.SF) and in ipsilateral sensorimotor ROIs (R.PrC and R.PoC). The latter was mainly mediated by ipsilateral visual and visuomotor ROIs (R.CU and R.PCU), by a contralateral sensorimotor ROI (L.PrC), and by the bilateral frontal ROIs (L.SF and R.SF). Moreover, the same ipsilateral sensorimotor areas (R.PrC and R.PoC) that were shown to be higher in degree also exhibited a significantly increased alpha-outflow mainly towards other areas in the same hemisphere (among them R.SP, R.PCU, R.SF).

As to the beta band (Figure 7), some visual ROIs (L.CU and R.CU) exhibited significantly higher beta-inflow and beta-outflow. The contralateral visuomotor ROI L.SP was characterized by a significantly decreased beta-inflow, especially from sensorimotor ROIs in the same hemisphere (L.PrC and L.PaC). Indeed, the latter ROIs, together with L.PoC, had decreased beta-outflow not only towards L.SP but, interestingly, also towards ipsilateral sensorimotor ROIs (R.PrC and R.PoC).

## 4. Discussion

This study investigates alpha and beta mechanisms related to the preparation of reaching movements by analyzing the cortical activity by means of event-related spectral perturbations, and the connectivity between regions by means of spectral Granger causality and graph analysis. The analysis of brain connectivity, either at rest or during a task, is today recognized as a fundamental tool to gain insights into how different brain regions work together (in a synergistic or antagonistic way) and exchange information to achieve behavior, and how this coordinated activity is disrupted in pathological states. Among the measures of connectivity, GC is a popular statistical method to analyze directed interactions in multivariate dynamical systems [58]. An attractive property of GC for brain connectivity investigation is its frequency domain formulation, eligible for the analysis of causal interactions in specific frequency bands and, thus, particularly relevant in the case of neuroelectric signals, extremely rich in oscillatory content. In the context of brain connectivity networks, graph theoretical approaches provide a powerful way to quantify the topological properties of the networks, inferring meaningful attributes that improve the understanding of connectivity patterns and of their functional roles [59].

The present study provides a novel contribution to the investigation of electromagnetic brain activity and connectivity in reaching tasks. To the best of our knowledge, this is the first time that directed connectivity and direction-sensitive indices derived from the graph theory, joined with ERSP analysis, are applied to investigate a large set of brain areas in the preparation phase of a reaching task, providing an enriched EEG characterization and interpretation of brain regions’ activation and of their causal interactions during reaching movement preparation. Specifically, here, both ERSP and spectral GC were analyzed on the cortical activity reconstructed from the EEG while healthy subjects prepared a reaching movement compared to a rest condition.

### 4.1. Event-Related Spectral Perturbations

Reaching movement preparation was associated to alpha-ERD in visual and visuomotor ROIs (and only one sensorimotor ROI). Even though alpha-ERD exhibited slightly stronger results in the contralateral hemisphere (e.g., for SP and PCU in Figure 4), no significant differences were observed between hemispheres. It is worth noticing that this result held also without averaging together different targets, i.e., by performing the ERSP analysis within each single target, as reported in Supplementary Figure S2. From this figure, no significant inter-hemispheric difference in the alpha band was observed, widely across ROIs and targets, except only for the target located most rightwards, that showed a stronger alpha-ERD for the contralateral side in PrC and PoC. Alpha-ERD is likely associated to the goal-directed visuomotor nature of the task, involving both visuo-spatial attention and the processing of spatial information to guide the hand to the proper position accurately (i.e., location of the target LED). As to the beta band, widespread beta-ERD was observed, stronger in sensorimotor and visuomotor ROIs, and significantly higher in the contralateral hemisphere in particular for the sensorimotor ROIs (this result held also within each target, see Supplementary Figure S3). These results on alpha- and beta-ERD agree with the study of Wang et al. [10], showing that during visually-cued movement preparation (even though finger movement and not reaching movement was analyzed), alpha-ERD was localized more posteriorly, while beta-ERD was more widespread and more lateralized. Considerations also come by looking at the alpha- and beta-ERSP dynamic in Figure 3. The visuomotor and mainly the sensorimotor ROIs exhibited time-increasing ERD (i.e., time-increasing disinhibition) in the alpha band and especially the beta band throughout the movement preparation period, with ERD further increasing during movement execution. This suggests a progressively growing engagement of sensorimotor ROIs during the entire trial, from movement preparation to execution. The same did not hold for visual ROIs (LO and CU). Indeed, after the transient related to visual-evoked potential, visual ROIs exhibited a constant alpha-band disinhibition in the preparation phase, suggesting a sustained visuo-spatial attention while preparing the action. Conversely, these ROIs tended to rapidly deactivate (i.e., ERSP tended to partially return towards 0) in the beta band, indicating a more marginal role in motor planning. Differences in alpha- and beta-ERD suggested that these rhythms are to some extent independent and with distinct functional relevance. Indeed, while the suppression of beta oscillations is tied to the activation of neuronal populations involved in movement, the suppression of alpha oscillations also reflects visual information processing and cognitive processing related to attention [60].

### 4.2. Connectivity Network and Centrality Indices

Causal influences between ROIs were analyzed via spectral GC and differences in connectivity network were assessed between reaching movement preparation and rest. In degree and out degree centrality indices were computed, quantifying overall connectivity inflow and outflow for each ROI, and were used to identify the ROIs that significantly exhibited changes in inward and outward connections during movement preparation compared to rest. It is worth noticing that, despite these differences being quantified by aggregating the information across reaching targets, the result was not driven by a small subset of targets, as the obtained differences were similar across targets (see Supplementary Figures S4–S7).

As to alpha-band connectivity, decreased interactions were mainly in visuomotor and visual regions, and were probably linked to the reduced amplitude of alpha oscillations in these ROIs (see alpha-ERD in Figure 2) and related to visual information processing (as decreased connectivity values can be related to desynchronization [35]). Interestingly, our results indicate a prevalent anterior-to-posterior direction of decreased alpha connections (from parietal to occipital and also from front-central to parietal and occipital regions), and with bilateral LO that most showed reduced alpha inflow. Previous studies have reported top-down modulatory influences in the alpha band from higher-level frontal and parietal areas to the lower-level visual cortex, as a mechanism for controlling visuo-spatial attention via facilitation (decreased alpha-band influences) and inhibition (increased alpha-band influences) [61,62]. Our findings appear in line with this hypothesis, with decreased inflow in the early visual cortex facilitating visual processing of stimuli for goal-directed movement. Decreased anterior-to-posterior alpha connectivity was accompanied by increased left-to-right alpha connections, and significantly higher alpha-inflow in ipsilateral sensorimotor ROIs. Importantly, the latter was also mediated by a contralateral sensorimotor ROI (L.PrC, see Figure 6b-left). Considering the inhibitory role of alpha rhythm, these results agree with previous evidence of inhibitory inter-hemispheric interactions between sensorimotor cortices [14] (concurring at facilitating the movement) that may be functionally implemented via alpha oscillations [10]. Furthermore, the inhibition of ipsilateral sensorimotor ROIs (R.PrC and R.PoC) was also exerted by a top-down mechanism operated by the two frontal ROIs (L.SF and R.SF, see Figure 6b-left), suggesting that top-down alpha influences from higher level areas can also be implicated in modulating (inhibiting or facilitating) motor-related processing other than sensory processing. Lastly, ipsilateral sensorimotor ROIs were also characterized by an increased alpha-outflow, mainly confined in the ipsilateral hemisphere (see Figure 6b-right), thus potentially contributing to further spreading and sustaining the inhibition in the ipsilateral hemisphere.

As to beta-band connectivity, a first notable result is the significantly higher beta-inflow and beta-outflow observed in visual ROIs (L.CU and R.CU). This might be related to ERD in the beta band that tended to reduce in the visual areas (ERSP tending to return closer to 0, Figure 3b upper panel) and that may be interpreted as an early disengagement of visual cortices from motor processing. Indeed, while visuomotor and sensorimotor ROIs likely contribute to motor-related processing in a sustained or increasing manner during the movement preparation period (as supported by their constant or increasing ERD dynamic, see Figure 3), it seems reasonable that visual cortices deactivate earlier. The second notable result is the significantly lower beta-outflow observed in contralateral sensorimotor ROIs (L.PrC, L.PoC, L.PaC) that had the main effect of significantly reducing beta-inflow in contralateral visuomotor ROIs (L.SP, Figure 7b-right). Based on this result, contralateral sensorimotor ROIs might act as hubs for beta-band desynchronization among movement-related regions, and such decoupling may represent a mechanism to interrupt the maintenance of the current motor output and favor regions to be engaged in the impending movement. Indeed, beta-band synchronization has been hypothesized to promote maintenance of the current sensorimotor state, while compromising the neural processing of new movements [63]. In this regard, it is also interesting to note that the two hemispheres are characterized by opposite changes in beta-band connections, with mainly increased beta-band connections entering and exiting from the ROIs in the ipsilateral hemisphere, as opposed to the contralateral hemisphere. This may indicate a coordinated beta-band competition between the two hemispheres, functionally relevant for performing unilateral movements.

As highlighted by our results, by analyzing the EEG via complementary investigations in the frequency domain (via ERSP and spectral GC, using also indices derived from graph theory), an enriched characterization of the preparation of reaching movements was provided. Overall, these findings substantiate the idea of the presence of different mechanisms during movement preparation operated by alpha and beta rhythms, comprising an alpha-mediated inhibition mechanism on the ipsilateral sensorimotor areas, and a beta-mediated disinhibition mechanism of the contralateral visuomotor and sensorimotor areas. Furthermore, alpha oscillations emerge as a general mechanism for inhibiting processing in task-irrelevant regions (alpha increase) and facilitating processing in task-relevant regions (alpha decrease), involving both motor and sensory regions. Conversely, beta-band desynchronization appears as a more motor-specific disinhibition mechanism; indeed, despite the widespread beta-ERD involving all regions, connectivity analysis reveals spatially specific differences in beta-band interactions where visual areas (although actively involved in sensory processing during the task) and also ipsilateral motor-related areas were characterized by beta-band connectivity increase. The present study not only contributes to expanding the neurophysiological description of motor-related mechanisms but may also have clinical and practical implications. For example, connectivity appears as a measure able to capture more subtle changes in brain functioning; thus, brain connectivity may provide markers of neuromotor disorders more sensitive to progression or improvement. Moreover, measures of connectivity have potential applications in motor-based brain–computer interfaces; indeed, motor states can be decoded exploiting artificial intelligence approaches not only by using scalp-level EEG [64,65], but also from features related to brain network connectivity [66]. Interestingly, the knowledge learned by these decoders could also be exploited to analyze, in a data-driven way, the most relevant interactions for a target variable under analysis [64,65,67,68,69] (e.g., a specific movement), by designing and applying explainable artificial intelligence approaches specific for functional connectivity analyses.

Of course, the present study has some limitations. First, our analyses were not conducted on the whole cortex parcellation but on a selection of ROIs known to be implicated in reaching movement preparation and control. However, other ROIs (not considered in the performed analyses) may also be involved, e.g., the right inferior frontal gyrus (R.IF), a region found to play a role in motor control via top-down inhibition of planned or ongoing action [70]. As complementary analyses, we also performed the same analyses conducted in this study for bilateral IF areas in Supplementary Figures S8 and S9. Here, the signal representative of L.IF and R.IF was obtained, for each hemisphere, by averaging the signals of the pars opercularis, pars orbitalis, and pars triangularis, since these three regions compose the inferior frontal gyrus in the Desikan–Killiany atlas, adopted in this study for cortex parcellation. As obtained for SF, IF (both left and right) had small and bilateral ERDs during movement preparation and was involved in the alpha-mediated top-down inhibition of the ipsilateral sensorimotor areas. Thus, future studies could benefit from considering the whole cortex parcellation to avoid missing potentially relevant ROIs. Furthermore, the selected ROIs were based on the Desikan–Killiany atlas, and some of them englobe large portions of the cortex. In particular, as concerning the sensorimotor areas, we did not specifically consider the primary motor cortex (M1), supplementary motor areas (SMA), and premotor cortex (PMC), which are small regions in the sensorimotor cortex, and deemed to be core motor areas, largely investigated in connectivity studies [23,34,35]. Rather, we preferred to consider larger areas (likely englobing the previous core areas) also due to the use of a template head model for cortical source estimation, rather than individual head models. The use of a template head model (which, however, is commonly adopted in the literature when individual brain MRIs are not available [71]), unavoidably leads to a reduction in spatial accuracy in source localization and spatial inaccuracy may have a greater effect when small areas consisting of a low number of voxels are selected. By considering the average behavior of larger areas, spatial inaccuracies may have a more tolerable impact. Another limitation is related to the adoption of a fixed and short time window for computing the spectral GC, i.e., 1 s windows for rest and 1 s windows for reaching preparation, concatenated across trials. Indeed, movement preparation is a dynamic process that could benefit from a dynamic description of connectivity between brain regions. Furthermore, more accurate results with parametric spectral GC are known to be obtained as the window length increases [58]. However, due to the trial-based nature of the experimental paradigm, the movement preparation phase was inherently limited. These aspects may be addressed in the near future by studying the dynamic of spectral GC via non-parametric methods.

## 5. Conclusions

In conclusion, in this study, we investigated the frequency-specific changes in cortical activity and in directed connectivity evoked by the preparation of a reaching task within a network of task-related areas, spanning from occipital to parietal and fronto-central cortices.

Our results suggest that alpha and beta oscillations are functionally involved in the preparation of reaching movements in different ways. That is, beta mainly reflects the disinhibition of areas involved in movement, mainly contralateral visuomotor and sensorimotor areas, and concurs at coordinating the disinhibition among these areas. Alpha also reflects visual processing and visuo-spatial attention and concurs at mediating an inhibition mechanism (inter-hemispheric and top-down) of the ipsilateral sensorimotor areas (to facilitate the preparation of the unilateral upcoming movement) and the disinhibition of visual cortices (to facilitate visuo-spatial attention during preparation).

Overall, this study contributes to enriching the description of the neural mechanisms underlying reaching movement preparation in healthy subjects, for a better comprehension of the neurophysiological correlates. In prospective, this knowledge could be useful to analyze alterations occurring in pathology (e.g., stroke) and to improve diagnostic and therapeutic applications.

## Data Availability

The data presented in this study are available on request from the corresponding author.

## Supplementary Materials

The following supporting information can be downloaded at: https://www.mdpi.com/article/10.3390/s23073530/s1, Figure S1: Alpha (a) and beta (b) event-related spectral perturbations (ERSPs) at Cz considering a larger epoch (from −1 to 11 s respect to the cue-onset) than the one used in the main text (from −1 to 3 s). The epoch considered here includes the overall trial, i.e., both the forward and backward movement, from 0 to 10 s; an additional final second is displayed (from 10 to 11 s), reporting also the first second of the random inter-trial interval (ranging from 2–3 s randomly) of the subsequent trial. This analysis was performed to observe the ERSP dynamic for a longer period, checking for ERSP rebound towards rest values (i.e., towards 0). Here, ERSPs were computed at the scalp level using the same procedure as at source-level (see Section 2.3.3) and are visualized as a function of time (as reported in Figure 3 at the source-level). Black tick line denotes the grand-average alpha-ERSP in the left plot and the grand-average beta-ERSP in the right plot, and shaded grey area denotes the standard error of the mean across subjects. The small black and purple triangles shown at the bottom of each plot mark the time associated to the cue onset and go onset of movements, respectively. The first purple triangle refers to the go-onset for the forward movement, while the second one refers to the backward movement. Figure S2. Target-specific alpha event-related spectral perturbations (ERSPs) during reaching movement preparation (*post-cue_late_* interval), in the different ROIs. Here, for each ROI, the alpha-ERSP was averaged within the second half of the movement preparation interval (from 1 to 2 s with respect to cue onset, i.e., *post-cue_late_* interval), separately for each target to reach. For each target and each ROI (grey: left ROI, red: right ROI), the bar height denotes the mean value across the subject and the error bar the standard error of the mean. The black square represents the rest position of the hand; the bar plots are topologically arranged inside the page according to the target position they refer to. The same statistical analyses as the ones conducted to produce Figure 4 in the main text were performed here; however, instead of performing comparisons based on *post-cue_late_* ERSP averaged across targets, the comparisons were performed separately for each target. Results of the performed statistical analyses are reported using symbols: symbols * (at the bottom of each panel) denote ERSPs significantly different compared to baseline (* p<0.05, ** p<0.01, *** p<0.001); symbols † (at the top of each panel) denote ROIs with significantly different ERSP between the left and right hemisphere († p<0.05, †† p<0.01, ††† p<0.001). Reported statistical results are corrected for multiple comparisons, with correction applied separately within each target. Note that significant inter-hemispheric difference was observed only in case of the most rightward target (bar plot at the bottom right), as to ROI PoC and PrC, with alpha-ERD significantly larger in the contralateral ROI than in the ipsilateral one. Figure S3. Target-specific beta event-related spectral perturbations (ERSPs) during reaching movement preparation (*post-cue_late_* interval). Here, for each ROI, the beta-ERSP was averaged within the second half of the movement preparation interval (from 1 to 2 s with respect to cue onset, i.e., *post-cue_late_* interval), separately for each target to reach. See the caption of Supplementary Figure S2 for further details. Note that significant inter-hemispheric difference was observed for several targets and ROIs (especially sensorimotor and visuomotor), with beta-ERD significantly larger in the contralateral ROI than in the ipsilateral one. This result is in agreement with results obtained collapsing together all targets (see Figure 4 in the main text). Figure S4. ROIs with a significantly different in degree between center-out reaching preparation and rest in the alpha band, separately for each target position. The black square represents the rest position and each in degree representation is topologically placed according to the target it refers to. The same statistical analysis as the one conducted to produce Figs. 6 and 7 in the main text was performed. However, instead of performing comparisons based on the connections averaged across targets, the comparisons were performed separately for each target. Circle size reflects the strength of the significance (small: p<0.05, medium p<0.01, large: p<0.001); red/blue circles denote an increased/decreased measure (in degree or out degree) during movement preparation compared to rest. Figure S5. ROIs with a significantly different out degree between center-out reaching preparation and rest in the alpha band, separately for each target position. The black square represents the rest position. See the caption of Supplementary Figure S4 for further details. Figure S6. ROIs with a significantly different in degree between center-out reaching preparation and rest in the beta band, separately for each target position. The black square represents the rest position. See the caption of Supplementary Figure S4 for further details. Figure S7. ROIs with a significantly different out degree between center-out reaching preparation and rest in the beta band, separately for each target position. The black square represents the rest position. See the caption of Supplementary Figure S4 for further details. Figure S8. Alpha (a) and beta (b) event-related spectral perturbations (ERSPs) of the inferior frontal (IF) gyrus. The grand-average alpha-ERSP and beta-ERSP is reported for the left (black tick lines) and right (red tick lines) hemisphere. Shaded areas denote the standard error of the mean across subjects (in grey for the left ROI, in red for the right ROI). The small black and purple triangles shown at the bottom of each plot mark the time associated to the cue onset and go onset of the center-out reaching movement, respectively. Figure S9. Directed connections between ROIs—as measured by the spectral Granger causality—that resulted significantly higher (in red) or lower (in blue) during reaching movement preparation compared to rest, in alpha (left) and beta (right) bands. Here, also the inferior frontal (IF) gyrus is added to the set of ROIs considered in the study (thus, here, 18 ROIs in total are considered). To improve readability, ROI labels are displayed on the cortex in the middle panel, separately from the other panels.
