# Supplementary material for: Modulations of Cortical Power and Connectivity in Alpha and Beta Bands during the Preparation of Reaching Movements
Source: Sensors (Basel). 2023 Mar 28;23(7):3530. doi: 10.3390/s23073530 (PMC10099070; doi:10.3390/s23073530)
Supplement: Supplementary file 1 [file sensors-23-03530-s001.zip › sensors-2277527-supplementary.pdf]

Supplementary Materials

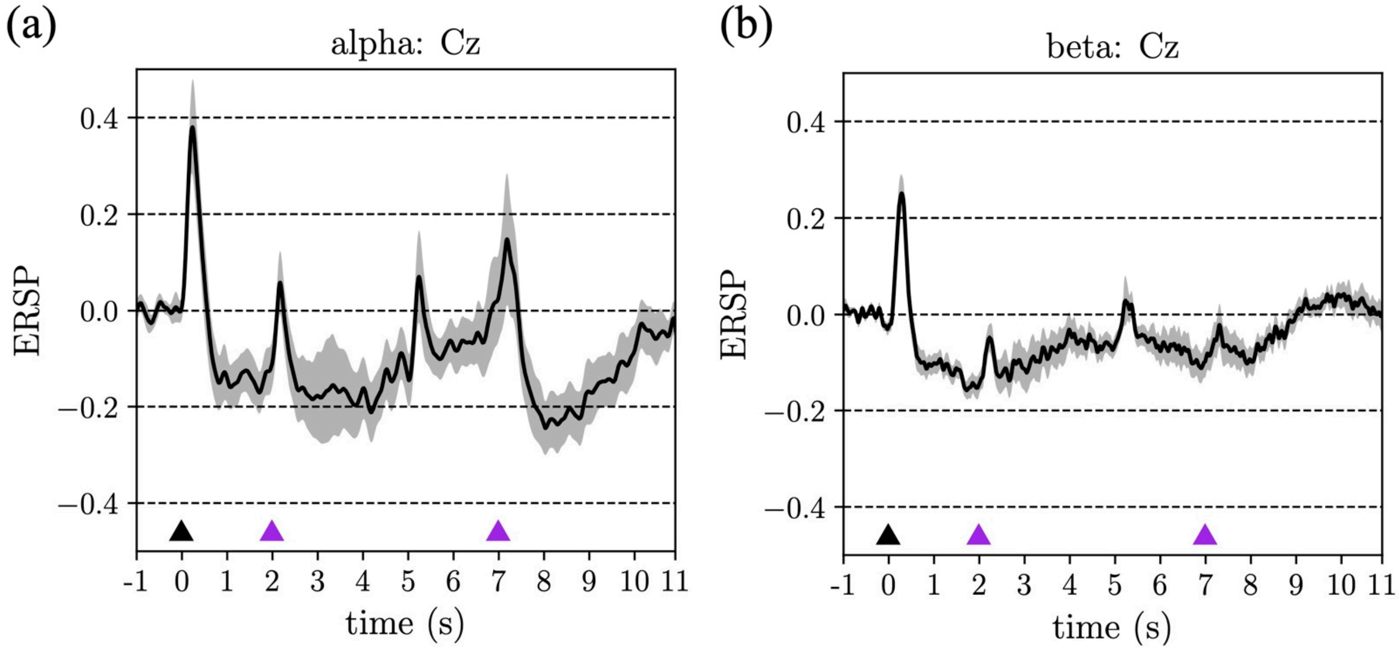

Supplementary Figure S1. Alpha (**a**) and beta (**b**) event-related spectral perturbations (ERSPs) at Cz considering a larger epoch (from -1 to 11 s respect to the cue-onset) than the one used in the main text (from -1 to 3 s). The epoch considered here includes the overall trial, i.e., both the forward and backward movement, from 0 to 10 s; an additional final second is displayed (from 10 to 11 s), reporting also the first second of the random inter-trial interval (ranging from 2-3 s randomly) of the subsequent trial. This analysis was performed to observe the ERSP dynamic for a longer period, checking for ERSP rebound towards rest values (i.e., towards 0). Here, ERSPs were computed at the scalp level using the same procedure as at source-level (see Section 2.3.3) and are visualized as a function of time (as reported in Figure 3 at the source-level). Black tick line denotes the grand-average alpha-ERSP in the left plot and the grand-average beta-ERSP in the right plot, and shaded grey area denotes the standard error of the mean across subjects. The small black and purple triangles shown at the bottom of each plot mark the time associated to the cue onset and go onset of movements, respectively. The first purple triangle refers to the go-onset for the forward movement, while the second one refers to the backward movement.

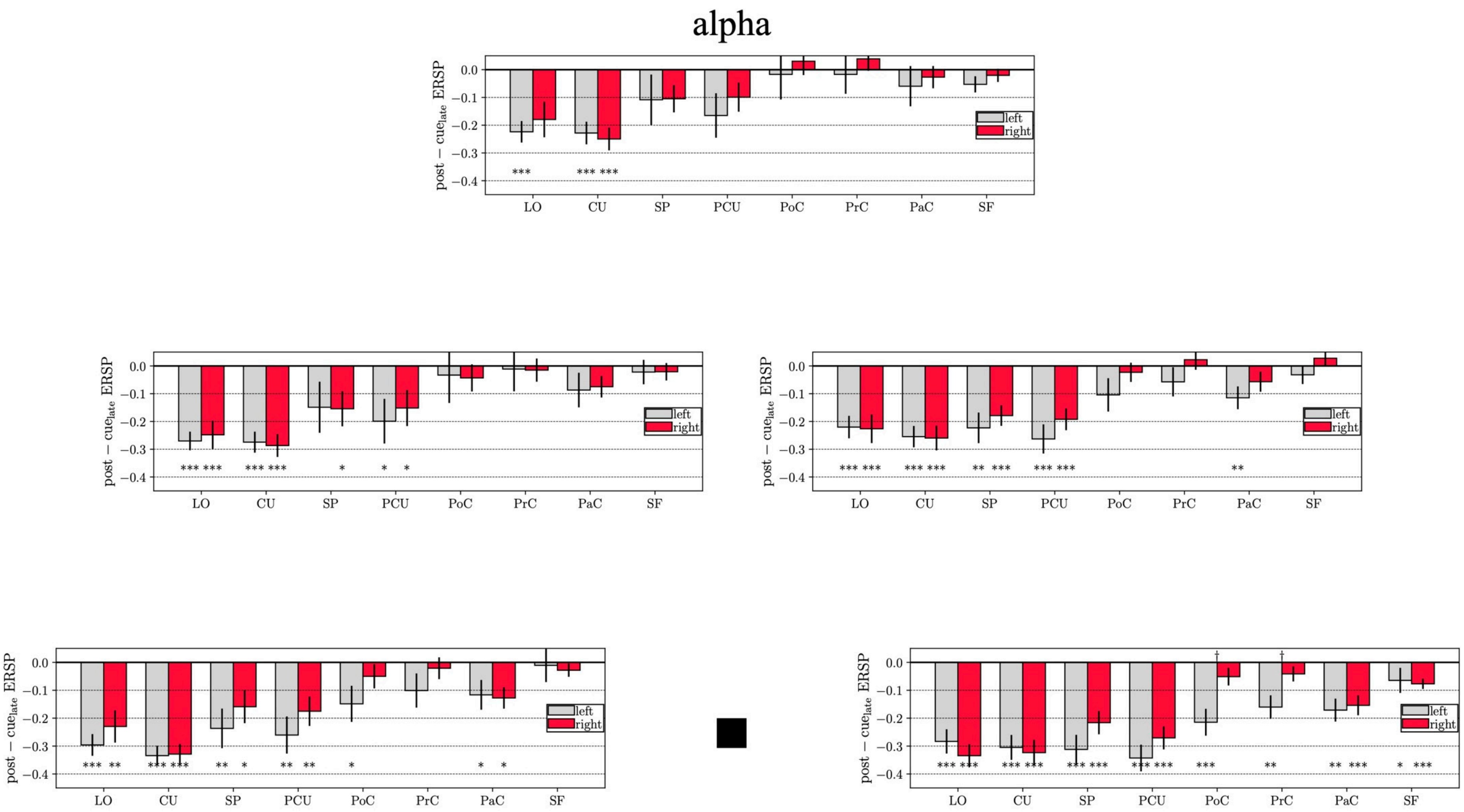

Supplementary Figure S2. Target-specific alpha event-related spectral perturbations (ERSPs) during reaching movement preparation (*post-cue$_{late}$* interval), in the different ROIs. Here, for each ROI, the alpha-ERSP was averaged within the second half of the movement preparation interval (from 1 to 2 s with respect to cue onset, i.e., *post-cue$_{late}$* interval), separately for each target to reach. For each target and each ROI (grey: left ROI, red: right ROI), the bar height denotes the mean value across the subject and the error bar the standard error of the mean. The black square represents the rest position of the hand; the bar plots are topologically arranged inside the page according to the target position they refer to. The same statistical analyses as the ones conducted to produce Figure 4 in the main text were performed here; however, instead of performing comparisons based on *post-cue$_{late}$* ERSP averaged across targets, the comparisons were performed separately for each target. Results of the performed statistical analyses are reported using symbols: symbols * (at the bottom of each panel) denote

ERSPs significantly different compared to baseline (* $p < .05$, ** $p < .01$, *** $p < .001$); symbols † (at the top of each panel) denote ROIs with significantly different ERSP between the left and right hemisphere († $p < .05$, †† $p < .01$, ††† $p < .001$). Reported statistical results are corrected for multiple comparisons, with correction applied separately within each target. Note that significant inter-hemispheric difference was observed only in case of the most rightward target (bar plot at the bottom right), as to ROI PoC and PrC, with alpha-ERD significantly larger in the contralateral ROI than in the ipsilateral one.

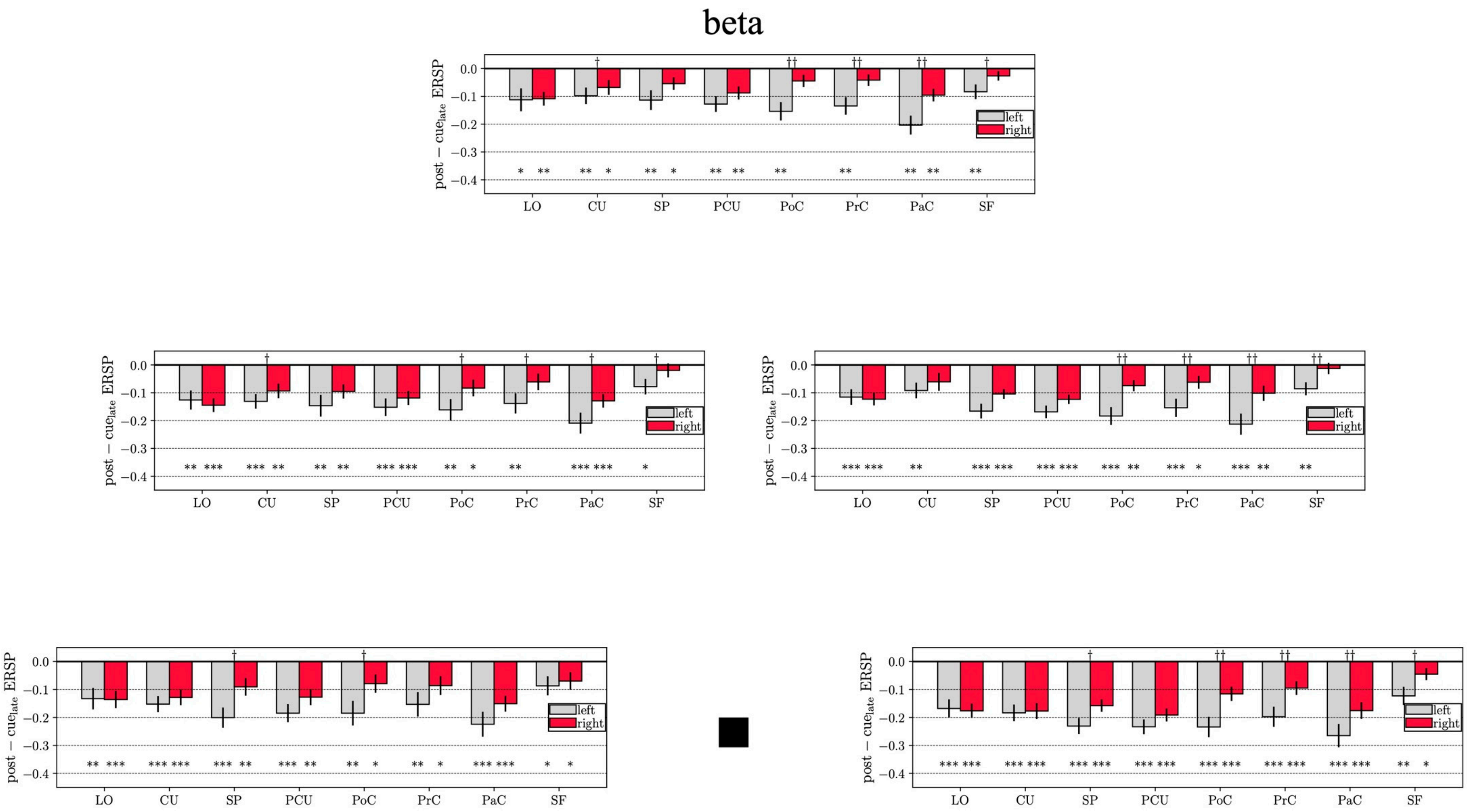

Supplementary Figure S3. Target-specific beta event-related spectral perturbations (ERSPs) during reaching movement preparation ($post\text{-}cue_{late}$ interval). Here, for each ROI, the beta-ERSP was averaged within the second half of the movement preparation interval (from 1 to 2 s with respect to cue onset, i.e., $post\text{-}cue_{late}$ interval), separately for each target to reach. See the caption of Supplementary Figure S2 for further details. Note that significant inter-hemispheric difference was observed for several targets and ROIs (especially sensorimotor and visuomotor), with beta-ERD significantly larger in the contralateral ROI than in the ipsilateral one. This result is in agreement with results obtained collapsing together all targets (see Figure 4 in the main text)

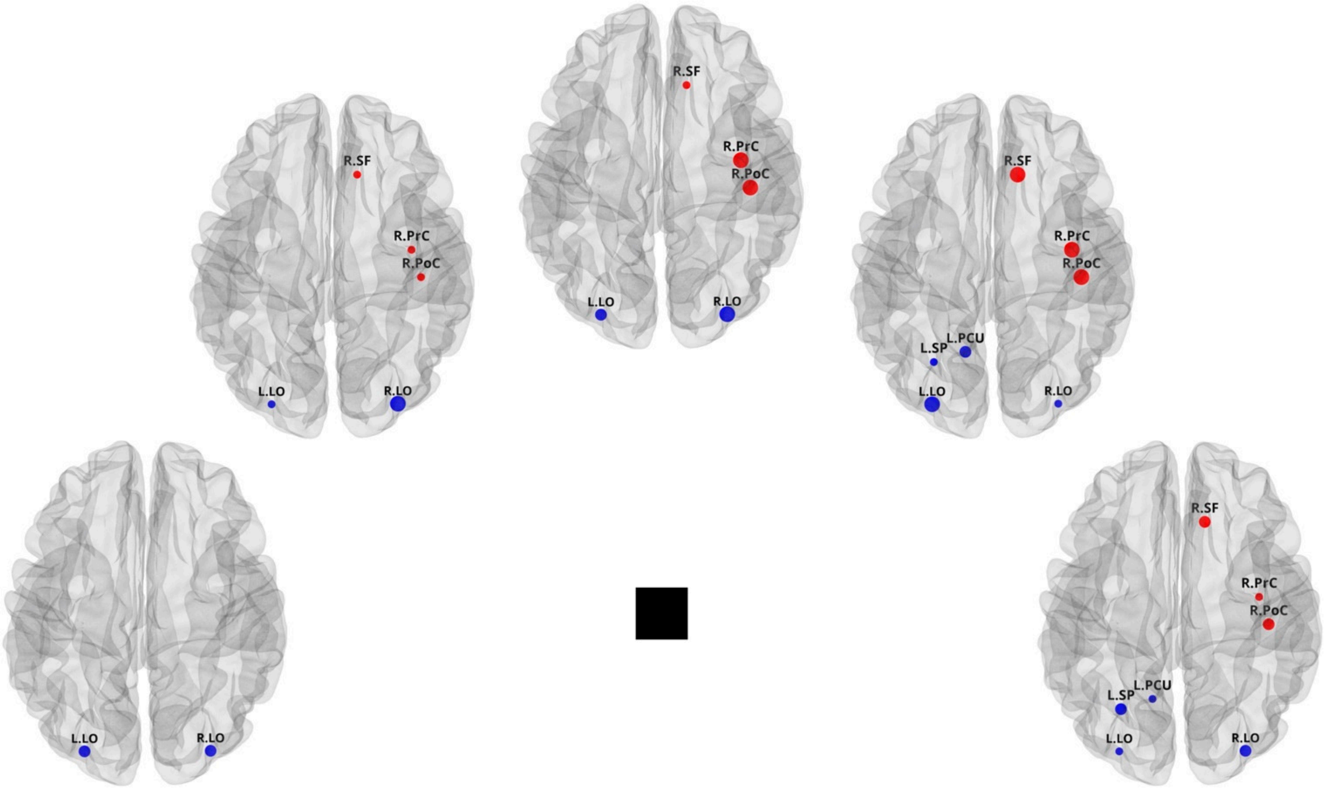

Supplementary Figure S4. ROIs with a significantly different in degree between center-out reaching preparation and rest in the alpha band, separately for each target position. The black square represents the rest position and each in degree representation is topologically placed according to the target it refers to. The same statistical analysis as the one conducted to produce Figs. 6 and 7 in the main text was performed. However, instead of performing comparisons based on the connections averaged across targets, the comparisons were performed separately for each target. Circle size reflects the strength of the significance (small: $p < .05$, medium $p < .01$, large: $p < .001$); red/blue circles denote an increased/decreased measure (in degree or out degree) during movement preparation compared to rest.

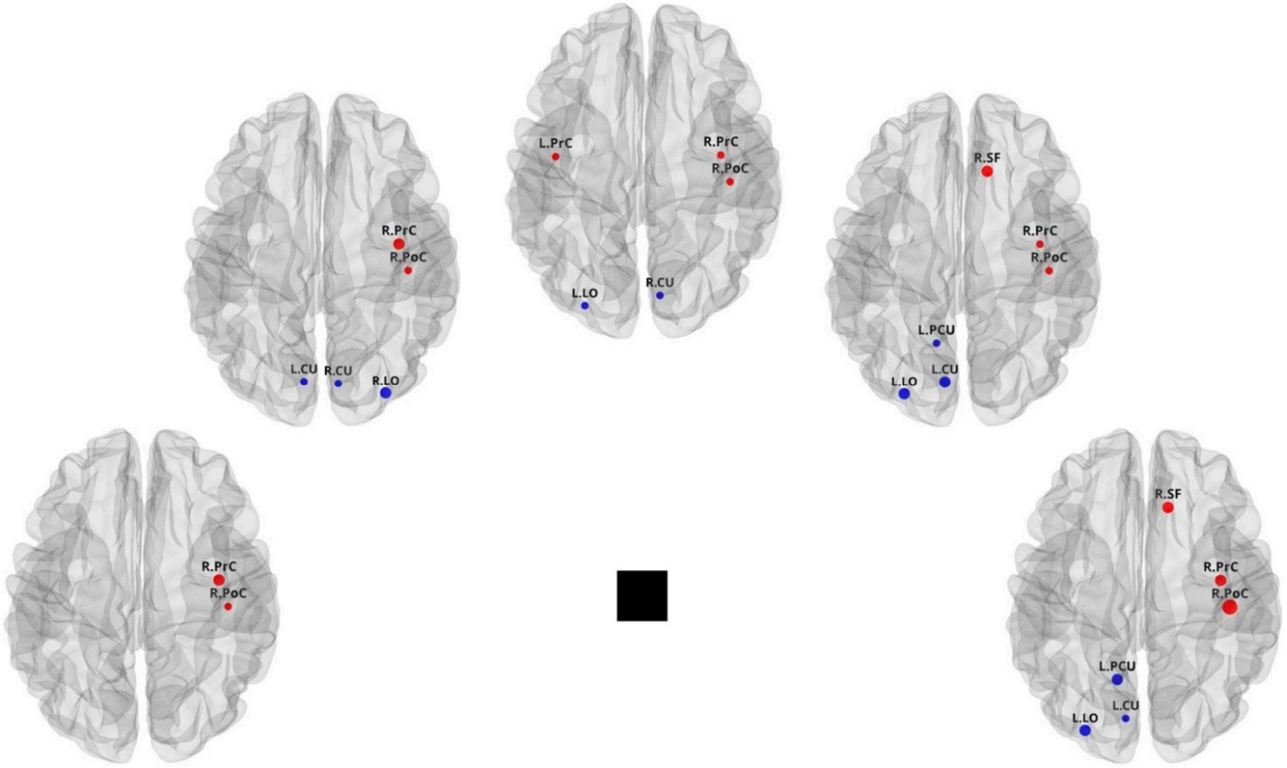

Supplementary Figure S5. ROIs with a significantly different out degree between center-out reaching preparation and rest in the alpha band, separately for each target position. The black square represents the rest position. See the caption of Supplementary Figure S4 for further details.

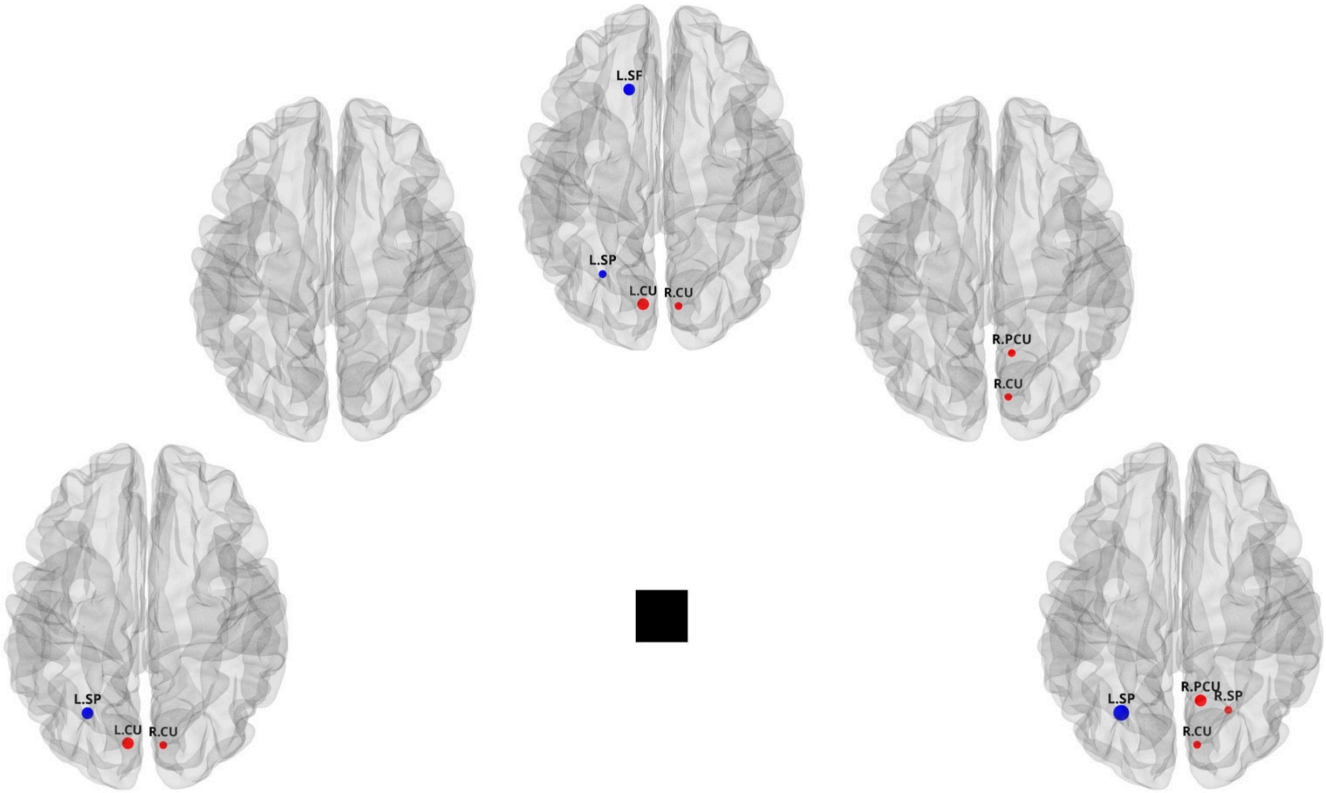

Supplementary Figure S6. ROIs with a significantly different in degree between center-out reaching preparation and rest in the beta band, separately for each target position. The black square represents the rest position. See the caption of Supplementary Figure S4 for further details.

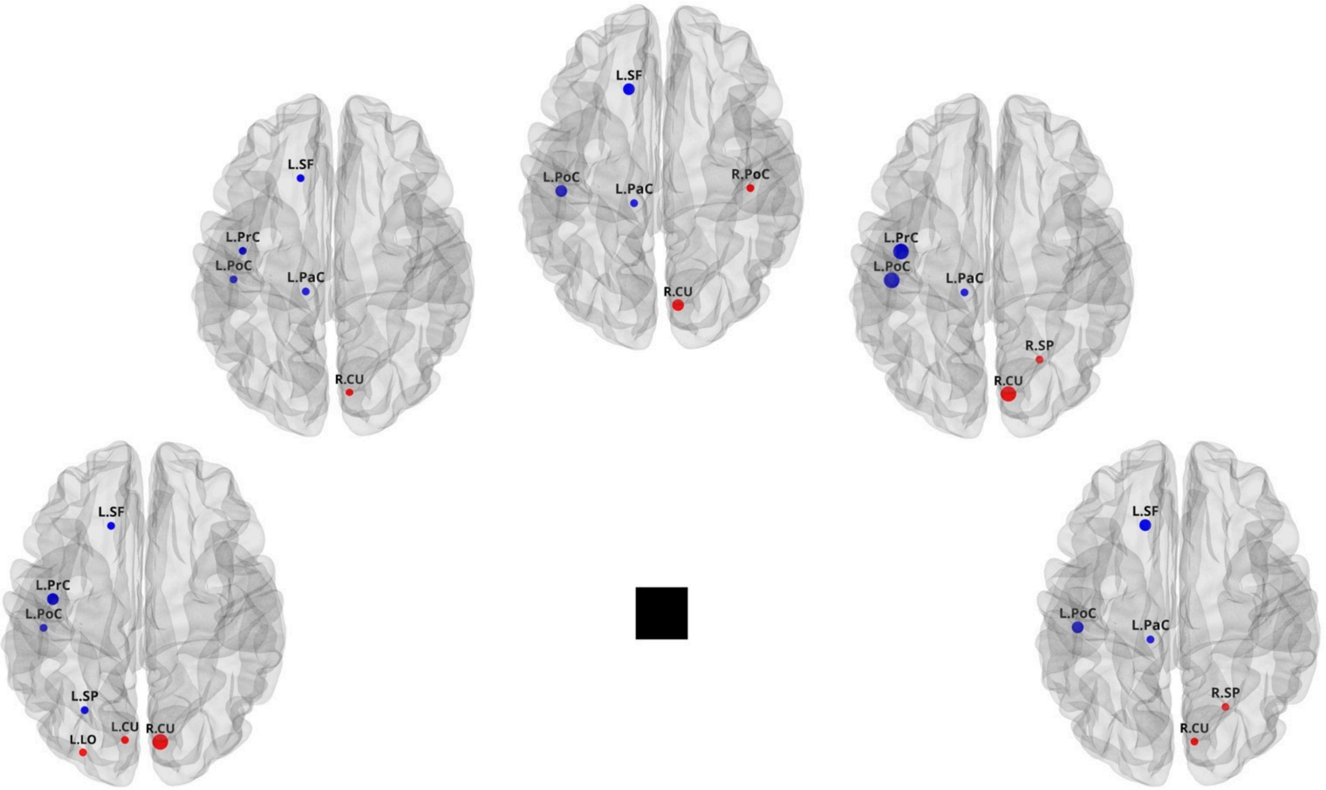

Supplementary Figure S7. ROIs with a significantly different out degree between center-out reaching preparation and rest in the beta band, separately for each target position. The black square represents the rest position. See the caption of Supplementary Figure S4 for further details.

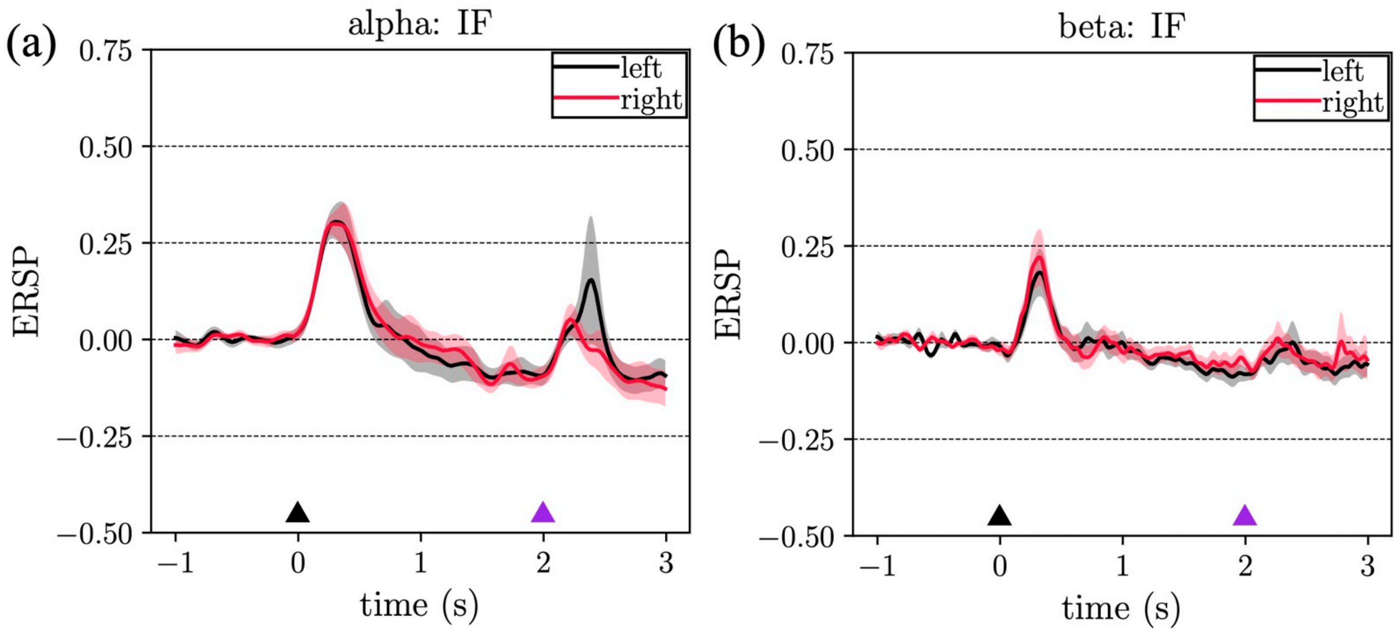

Supplementary Figure S8. Alpha (**a**) and beta (**b**) event-related spectral perturbations (ERSPs) of the inferior frontal (IF) gyrus. The grand-average alpha-ERSP and beta-ERSP is reported for the left (black tick lines) and right (red tick lines) hemisphere. Shaded areas denote the standard error of the mean across subjects (in grey for the left ROI, in red for the right ROI). The small black and purple triangles shown at the bottom of each plot mark the time associated to the cue onset and go onset of the center-out reaching movement, respectively.

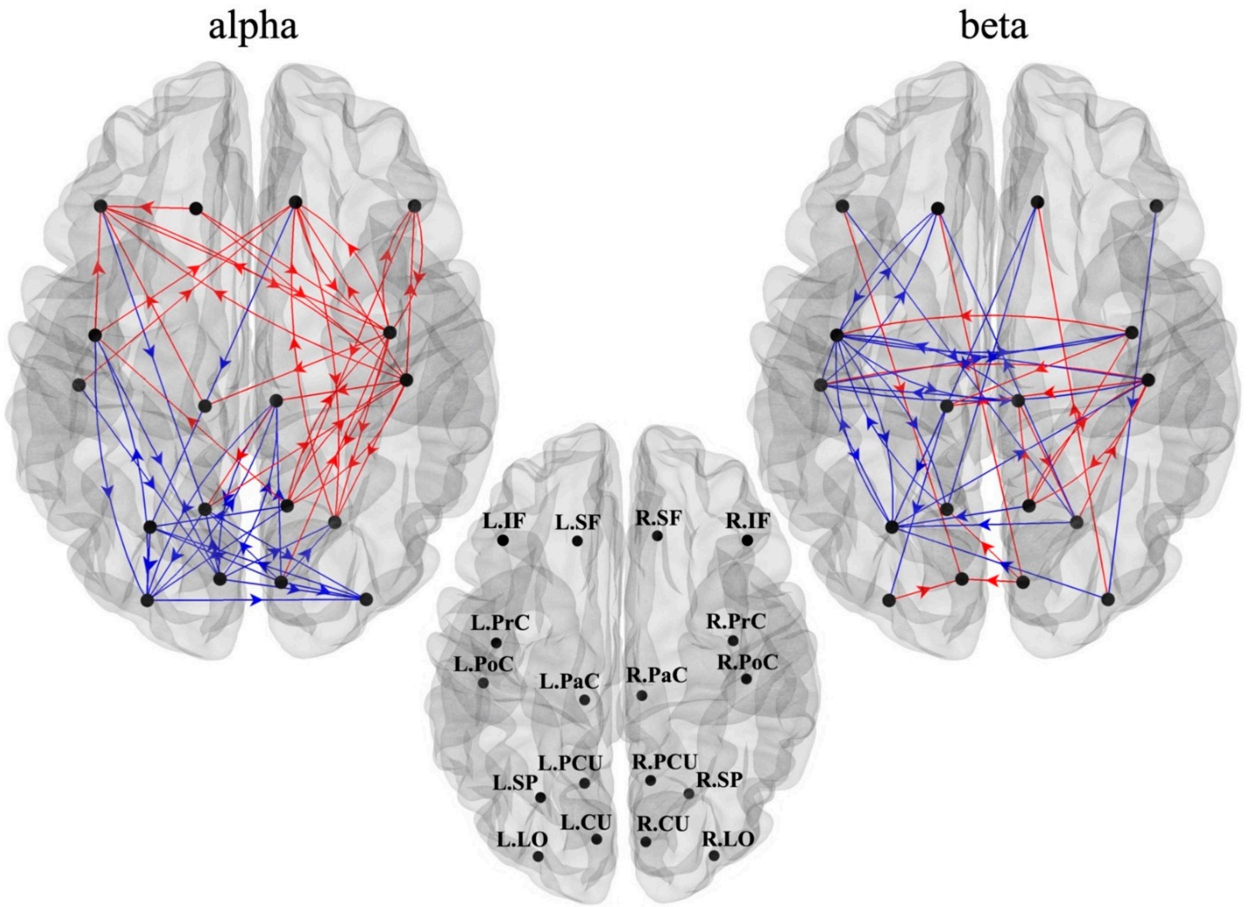

Supplementary Figure S9. Directed connections between ROIs – as measured by the spectral Granger causality – that resulted significantly higher (in red) or lower (in blue) during reaching movement preparation compared to rest, in alpha (**left**) and beta (**right**) bands. Here, also the inferior frontal (IF) gyrus is added to the set of ROIs considered in the study (thus, here, 18 ROIs in total are considered). To improve readability, ROI labels are displayed on the cortex in the middle panel, separately from the other panels.
